# Laparoscopic IPOM versus open sublay technique for elective incisional hernia repair: a registry-based, propensity score-matched comparison of 9907 patients

**DOI:** 10.1007/s00464-018-06629-2

**Published:** 2019-01-02

**Authors:** F. Köckerling, T. Simon, D. Adolf, D. Köckerling, F. Mayer, W. Reinpold, D. Weyhe, R. Bittner

**Affiliations:** 1 Department of Surgery, Center for Minimally Invasive Surgery, Academic Teaching Hospital of Charité Medical School, Vivantes Hospital, Neue Bergstrasse 6, 13585 Berlin, Germany; 2 Department of General and Visceral Surgery, GRN – Hospital Weinheim, Röngtenstraße 1, 69469 Weinheim, Germany; 3 StatConsult GmbH, Halberstädter Straße 40 a, 39112 Magdeburg, Germany; 4 Imperial College School of Medicine, South Kensington Campus, SW7 2A2 London, UK; 5 Department of Surgery, Paracelsus Medical University, Müllner Hauptstrasse 48, 5020 Salzburg, Austria; 6 Department of Surgery, Wilhelmsburger Hospital Groß Sand, Academic Teaching Hospital of University Hamburg, Groß Sand 3, 21107 Hamburg, Germany; 7 Department of General and Visceral Surgery, Pius Hospital, University Hospital of Visceral Surgery, Georgstraße 12, 26121 Oldenburg, Germany; 8 Winghofer Medicum Hernia Center, Winghofer Straße 42, 72108 Rottenburg am Neckar, Germany

**Keywords:** Incisional hernia, Laparoscopic IPOM, Sublay, Complications, Hernia registry

## Abstract

**Background:**

For comparison of laparoscopic IPOM versus sublay technique for elective incisional hernia repair, the number of cases included in randomized controlled trials and meta-analyses is limited. Therefore, an urgent need for more comparative data persists.

**Methods:**

In total, 9907 patients with an elective incisional hernia repair and 1-year follow-up were selected from the Herniamed Hernia Registry between September 1, 2009 and June 1, 2016. Using propensity score matching, 3965 (96.5%) matched pairs from 4110 laparoscopic IPOM and 5797 sublay operations were formed for comparison of the techniques.

**Results:**

Comparison of laparoscopic IPOM versus open sublay revealed disadvantages for the sublay operation regarding postoperative surgical complications (3.4% vs. 10.5%; *p* < 0.001), complication-related reoperations (1.5% vs. 4.7%; *p* < 0.001), and postoperative general complications (2.5% vs. 3.7%; *p* = 0.004). The majority of surgical postoperative complications were surgical site infection, seroma, and bleeding. Laparoscopic IPOM had disadvantages in terms of intraoperative complications (2.3% vs. 1.3%; *p* < 0.001), mainly bleeding, bowel, and other organ injuries. No significant differences in the recurrence and pain rates at 1-year follow-up were observed.

**Conclusion:**

Laparoscopic IPOM was found to have advantages over the open sublay technique regarding the rates of both surgical and general postoperative complications as well as complication-related reoperations, but disadvantages regarding the rate of intraoperative complications.

Management pattern for ventral and incisional hernias are heterogeneous, often with little supporting evidence or correlation with existing evidence [1]. In a systematic review and network meta-analysis of mesh location in open ventral hernia repair, sublay mesh location had lower complication rates than other mesh locations [2]. An expert consensus endorsed sublay as the optimal mesh location in open, elective ventral hernia repair [1]. Numerous meta-analyses demonstrated that laparoscopic incisional and ventral hernia repair is a feasible and effective alternative to the open technique and is associated with lower incidence of wound complications [3–8]. In these meta-analyses comparing laparoscopic versus open repair of ventral hernias, data on primary hernias (umbilical, epigastric) and secondary (incisional) hernias were pooled [9]. As treatment outcomes of primary and incisional ventral hernias show significant differences, it is essential to conduct studies that compare the various surgical techniques focusing on a single hernia type [9–12]. Two meta-analyses compared laparoscopic and open techniques in incisional hernia repair only [13–16].

Based on six randomized controlled trials (RCTs) with a maximum of 751 patients, the largest of those two meta-analyses found a statistically significant reduction in wound complications with laparoscopic compared to open repair of incisional hernias [13–16]. However, the rate of bowel complications was significantly higher for the laparoscopic approach [14].

On the basis of these findings international guidelines recommend laparoscopic over open repair of incisional hernias due to the significantly reduced risk of wound complications, provided that the higher risk of intraoperative complications has been carefully evaluated [17–21].

On the contrary, a nationwide study of the Danish Hernia Database on early outcomes after incisional hernia repair observed major complications in 2.8% of open and in 4.8% of laparoscopic repairs with a total morbidity rate of 10.1% in open and 11.8% in laparoscopic repairs [22]. These findings indicate that outcomes after incisional hernia repair, particularly concerning the laparoscopic approach, are unsatisfactory [22].

The current analysis used prospective data from the Herniamed Hernia Registry to compare outcomes for the laparoscopic intraperitoneal onlay mesh (IPOM) and open sublay techniques recommended in the guidelines for incisional hernia repair. Propensity score (PS) matching was utilized for statistical analysis of the prospective data [23]. Analyzed outcome variables included perioperative complications and complication-related reoperations, as well as the rates of recurrence, pain at rest and on exertion, and pain requiring treatment after 1-year follow-up.

## Methods

The Herniamed quality assurance study is a multicenter, internet-based hernia registry [24–27] with voluntary participating institutions which incorporates prospective data of patients who have undergone routine hernia surgery. These data are obtained from 618 voluntarily participating hospitals and surgeons engaged in private practice (Herniamed Study Group) mainly in Germany, Austria, and Switzerland (status: July 3rd, 2017). In Germany, surgeons in private practice are not employed by a hospital [27]. Rather, they operate on patients in outpatient/ambulatory surgical centers or hospitals for a fee [27]. All patients gave informed consent agreeing to participate. As part of the informed consent declaration, information provided to patients regarding participation in the Herniamed Registry included the request that the hospital or medical practice providing treatment would like to be informed about any problem occurring after the operation and that patients have the opportunity to attend clinical examination [27]. All postoperative complications occurring up to 30 days after surgery are recorded [27]. Postoperative pain at 1 week or 1 month and quality of life are not collected in the registry. At 1-year follow-up, postoperative complications are once again reviewed when the general practitioner and patient are asked to report any recurrences, pain at rest, pain on exertion, and chronic pain requiring treatment [27]. If recurrence or chronic pain is reported by the patient or the general practitioner the patient can be requested to present themselves for clinical or radiological examination [27]. A recent publication has provided impressive evidence of the role of patient-reported outcomes for both recurrence and chronic pain following incisional hernia repair [28].

In the current analysis, prospective data of patients who underwent primary elective incisional hernia repair with the laparoscopic IPOM or open sublay approach were evaluated to compare both techniques with respect to perioperative and 1-year follow-up outcomes.

**Fig. 1:**
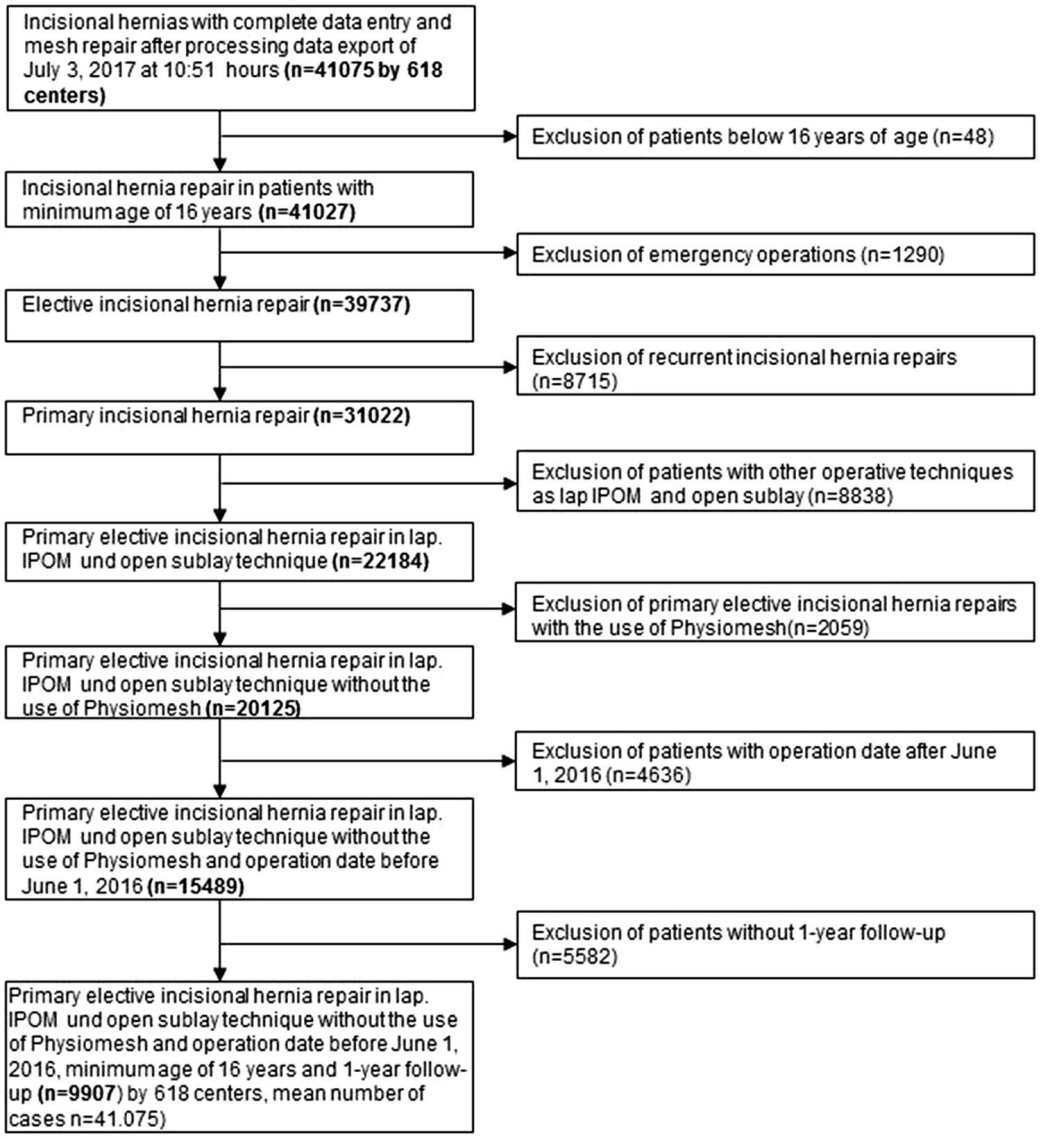
Flowchart of patient inclusion

The main inclusion criteria were minimum age of 16 years, primary elective incisional hernia repair using the laparoscopic IPOM or open sublay technique, no use of a Physiomesh [26], and availability of data at 1-year follow-up (Fig. 1). 9907 of 15,489 patients fulfilled these inclusion criteria (Fig. 1).

Physiomesh was excluded from this analysis, because Ethicon initiated a voluntary market withdrawal of Physiomesh in response to reports from the Danish Hernia Data Base and the Herniamed Hernia Registry about significantly higher recurrence rates in laparoscopic IPOM compared with other meshes [26].

For uniformity of the analyzed patient population, recurrent incisional hernias were also excluded.

**Fig. 2:**
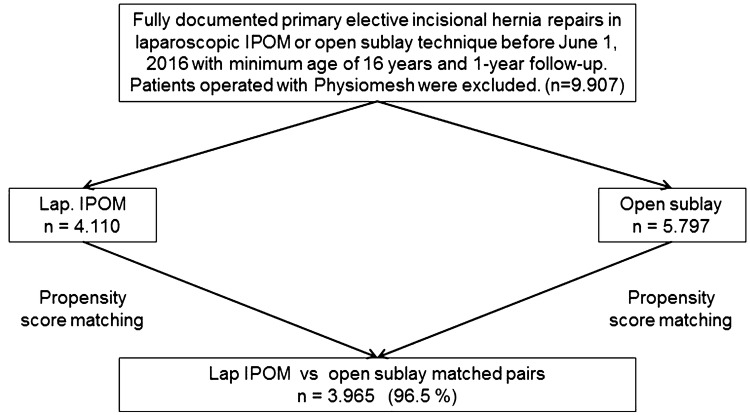
Flowchart of patient matching

In total, 9907 patients were selected between September 1, 2009 and June 1, 2016. Of these patients, 5797 had undergone open sublay and 4110 laparoscopic IPOM operations (Fig. 2).

Pairwise PS matching analysis was performed for these 9907 patients to obtain homogeneous comparison groups. For the purpose of the current analysis, the mutually independent matching groups laparoscopic IPOM versus open sublay (*n* = 3965; 96.5%) were thus formed (Fig. 2).

All statistical analyses were performed using the Software SAS 9.4 (SAS Institute Inc., Cary, NC) and intentionally calculated to a full significance level of 5%, that is, they were not corrected with respect to multiple tests, and each *p* ≤ 0.05 represents a significant result. Sole exception is the post hoc analysis of single items of intraoperative and postoperative complications. Here, a Bonferroni adjustment with factor 8 and 6, respectively, is performed.

Perioperative and 1-year follow-up outcomes (intra- and postoperative complications, complication-related reoperations, pain at rest and on exertion, pain requiring treatment, and recurrences at 1-year follow-up) were compared for laparoscopic IPOM versus open sublay using, first of all, PS matching. Matched samples were then analyzed via McNemar’s test. The obtained results are presented as the non-diagonal elements of the 2 × 2 frequency table, the corresponding *p* values and the odds ratio (OR) estimates with 95% confidence interval for matched samples.

Propensity score matching was performed using greedy algorithm and a caliper of 0.5 standard deviations. The variables used for matching were as follows: age (years), sex (male/female), body mass index (BMI) (kg/m^2^), American Society of Anesthesiologist (ASA) score (I–IV), preoperative pain (yes/no/unknown), defect size [European Hernia Society (EHS)] classification [29] [W1 = width < 4 cm, W2 = width ≥ 4–10 cm, W3 = width > 10 cm], defect localization (EHS classification medial, lateral, combined [29]), and presence of at least one risk factor (diabetes, chronic obstructive pulmonary disease, smoking, immunosuppression, aortic aneurysm, coagulopathy, corticosteroid therapy, antiplatelet, or anticoagulation therapy). The balance of the matched sample was assessed using standardized differences (also given for the pre-matched sample), which should not exceed 10% (< 0.1) after creating matched pairs.

For pairwise comparison of matching parameters between operation methods (in order to present the differences between the original pre-matched samples), *X*^2^ test and *t* tests (Satterthwaite) were performed for categorical and continuous variables, respectively.

## Results

Prior to PS matching, comparison of matching variables between laparoscopic IPOM and open sublay cohorts revealed statistically significant differences in age (*p* = 0.013), BMI, defect size, risk factors (*p* < 0.001 each), and EHS classification (*p* = 0.003). For example, compared with their laparoscopic IPOM counterparts, patients in the open sublay group had a significantly older age (mean age lap. IPOM 63.2 ± 12.8 years vs. sublay 63.8 ± 12.7 years), but lower BMI (mean BMI lap. IPOM 29.7 ± 5.7 vs. sublay 29.0 ± 5.6). Furthermore, the open sublay cohort had a significantly lower proportion of small defects (W1 < 4 cm lap. IPOM 36.0% vs. sublay 23.6%), a higher proportion of medial (EHS medial lap. IPOM 72.8% vs. sublay 74.7%), but lower proportion of combined defect localizations as per the EHS classification (EHS combined lap. IPOM 9.93% vs. sublay 7.95%) and a higher proportion of patients with risk factors (risk factors lap. IPOM 40.0% vs. sublay 44.0%).

PS matching was applied to match the 4110 patients who had undergone laparoscopic IPOM with the 5797 patients operated on with the open sublay technique. PS matching was applied to match the laparoscopic IPOM cohort (*n* = 4110) with the open sublay cohort (*n* = 5797).

Matching with the open sublay population was successfully applied for *n* = 3965 (96.5%) of the laparoscopic IPOM patients (Fig. 2).

In this matched sample with regards to the laparoscopic IPOM approach, the most frequently employed meshes (≥ 2%) were Parietex composite (27.2%), DynaMesh IPOM (21.1%), Parietene composite (9.1%), Parietex composite optimized (7.7%), Symbotex composite (5.3%), and TiMesh (5.2%). With the open sublay technique, the most frequently used meshes (≥ 2%) were Ultrapro (33.0%), Parietene ProGrip (7.0%), Parietex ProGrip (6.5%), Optilene Elastic (5.1%), Parietene light (4.7%), DynaMesh CICAT (4.6%), Prolene (3.9%), and TiMesh (2.2%).

Mesh fixation in the laparoscopic IPOM group was performed with tacker only in 55.9%, with tacker and suture in 36.6%, with suture alone in 4.3%, and other techniques in 3.2%. In the sublay group for mesh fixation in 78.6% only sutures, in 13.7% self-fixation, in 3.5% glue, in 3.6% suture and glue, and 0.6% other techniques were used. Defect closure in the laparoscopic IPOM group is only documented in 24.1% of the cases.

**Fig. 3:**
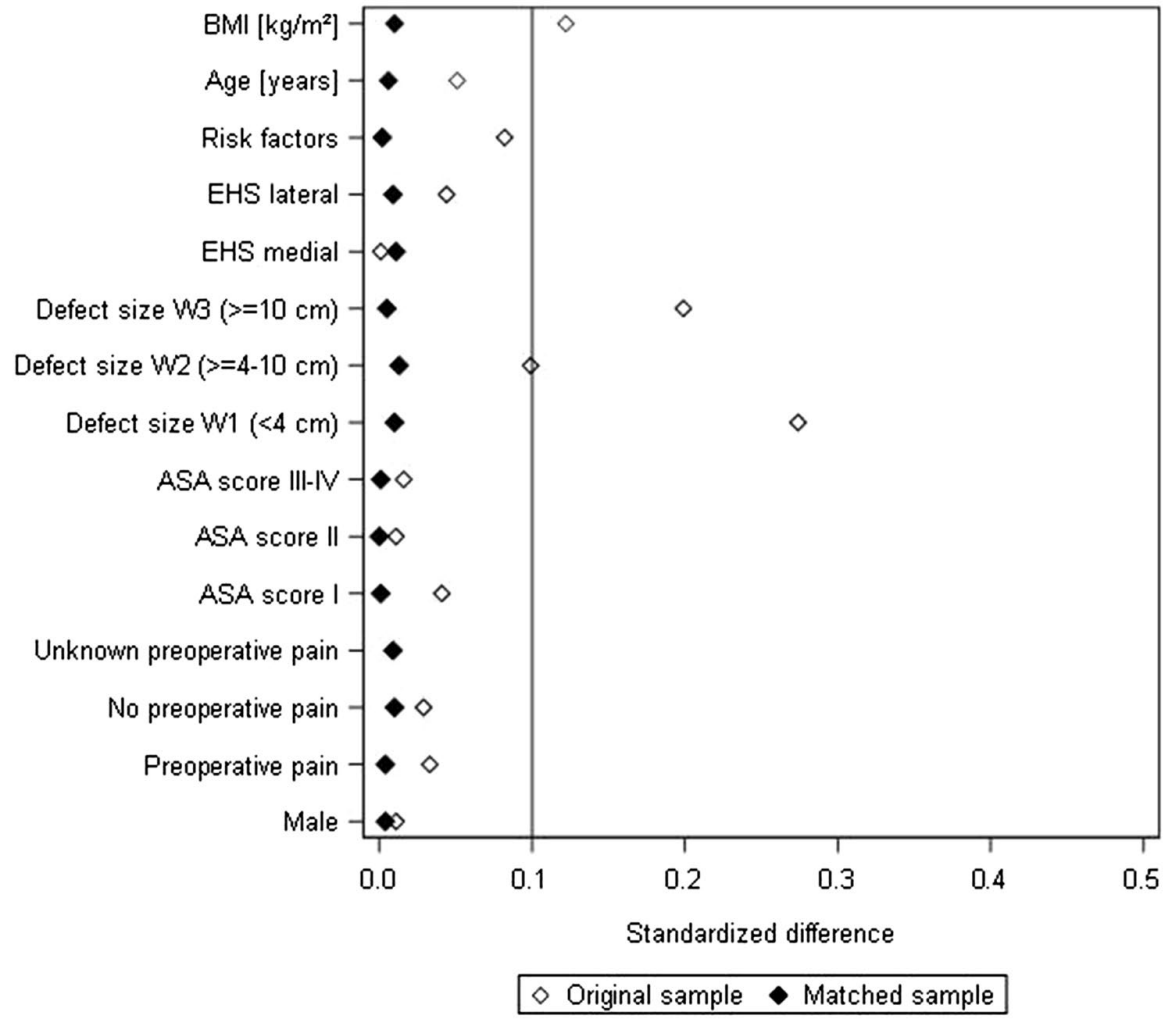
Standard differences between the matching variables both before (original sample) and after matching (matched sample)

Figure 3 illustrates the standard differences between matching variables, both before (original sample) and after matching. Notably, the standardized differences before matching were already relatively small, thus affirming that the discrepancies in baseline characteristics of the two cohorts were not extreme.

**Fig. 4:**
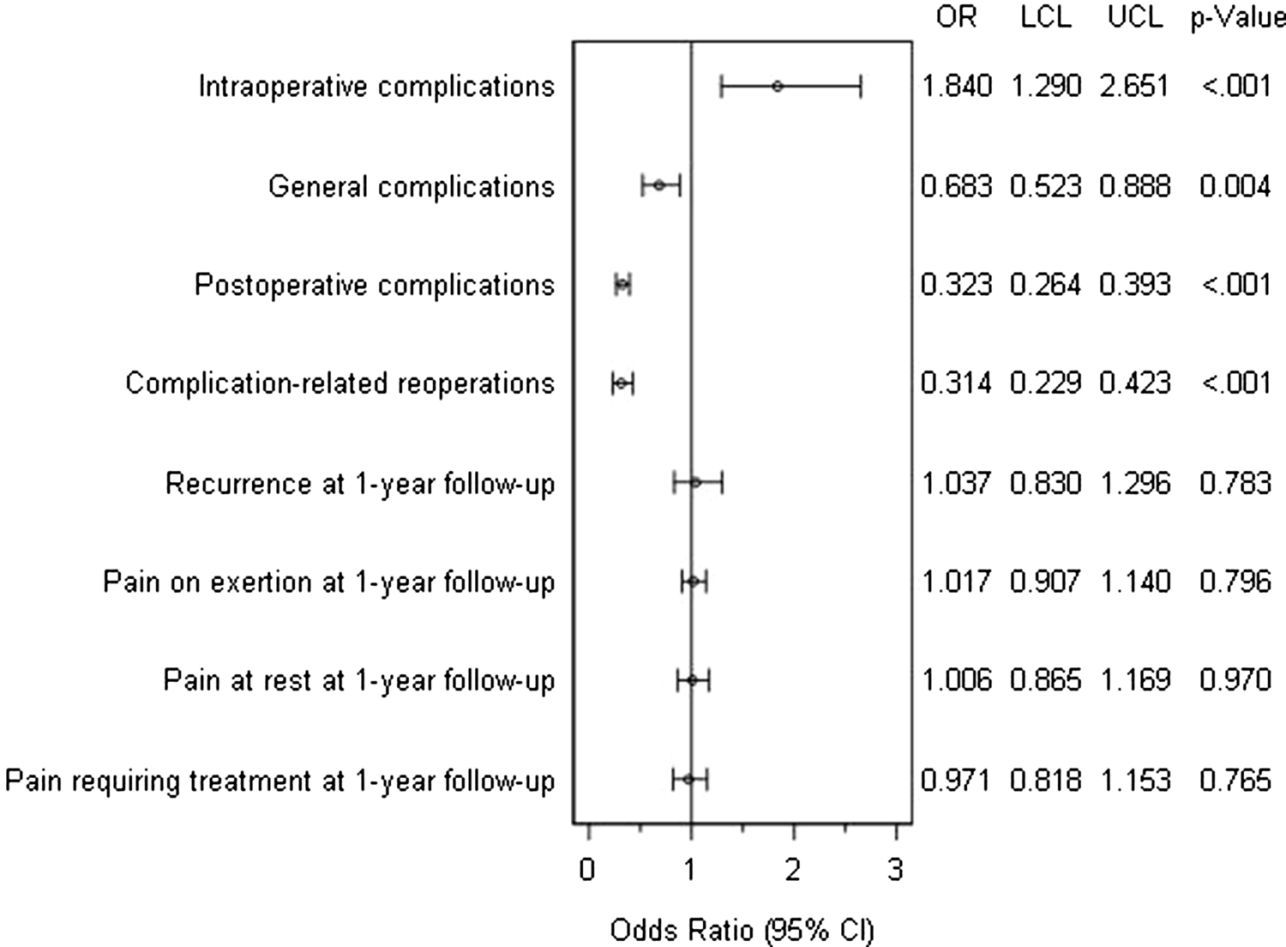
Results of matched pairs analysis of laparoscopic intraperitoneal onlay mesh versus open sublay in incisional hernia repair

Figure 4 summarizes the results of matched pairs analysis for laparoscopic IPOM versus open sublay, with respect to the various outcome parameters. Comparing the two surgical techniques, no statistically significant, systematic deviation was noted for recurrences [lap. IPOM 4.2% vs. sublay 4.1%, OR 1.037, 95% CI (0.830–1.296); *p* = 0.783], pain at rest [lap. IPOM 8.9% vs. sublay 8.9%; OR 1.006, 95% CI (0.865–1.169); *p* = 0.970], pain on exertion [lap. IPOM 15.4% vs. sublay 15.1%; OR 1.017, 95% CI (0.907–1.140); *p* = 0.796], and pain requiring treatment [lap. IPOM 6.8% vs. sublay 7.0%; OR 0.971, 95% CI (0.818–1.153); *p* = 0.765] after 1-year follow-up.

However, a significant deviation was observed to the disadvantage of the open sublay operation regarding the rate of surgical postoperative complications [lap. IPOM 3.4% vs. sublay 10.5%; OR 0.323, 95% CI (0.264–0.393); *p* < 0.001] (Table 1), mainly surgical site infection, seroma and bleeding (Table 2), complication-related reoperations [lap. IPOM 1.5% vs. sublay 4.7%; OR 0.314, 95% CI (0.229–0.423); *p* < 0.001), and general postoperative complications [lap. IPOM 2.5% vs. sublay 3.7%; OR 0.683, 95% CI (0.523–0.888); *p* = 0.004]. The complication-related reoperation rate for postoperative bleeding only showed also a significant deviation to the disadvantage of sublay repair [lap. IPOM 0.45% vs. sublay 1.6%; OR 0.281, 95% CI (0.124–0.579); *p* = 0.001].

**Table 1:** Results of matched pairs analysis in percentage

	Disadvantage		*p* value	OR for matched samples		
	Lap. IPOM	Open sublay		OR		
Intraoperative complication	2.32	1.26	< 0.001	1.840	1.290	2.651
General complication	2.50	3.66	0.004	0.683	0.523	0.888
Postoperative complication	3.38	10.47	< 0.001	0.323	0.264	0.393
Reoperation	1.46	4.67	< 0.001	0.314	0.229	0.423
Recurrence on 1-year follow-up	4.21	4.06	0.783	1.037	0.830	1.296
Pain on exertion on 1-year follow-up	15.36	15.11	0.796	1.017	0.907	1.140
Pain in rest on 1-year follow-up	8.90	8.85	0.970	1.006	0.865	1.169
Pain requiring treatment on 1-year follow-up	6.78	6.99	0.765	0.971	0.818	1.153

**Table 2:** Details of intra- and postoperative complications

	Disadvantage		p-value*	OR* for matched samples		
	Laparoscopic	Open		OR		
Intraoperative complications						
Bleeding	1.01	0.18	< 0.001	5.714	1.957	23.054
Injuries total	1.82	1.13	0.127	1.600	0.942	2.773
Vessels	0.40	0.00	–	–	–	–
Stomach	0.03	0.03	1.000	1.000	0.002	638.500
Bowel	1.08	0.86	1.000	1.265	0.660	2.460
Liver	0.00	0.03	–	–	–	–
Spleen	0.05	0.05	1.000	1.000	0.024	42.141
Bladder	0.10	0.13	1.000	0.800	0.083	6.603
Others	0.20	0.18	1.000	1.143	0.236	5.832
Postoperative complications						
Bowel injury	0.40	0.25	1.000	1.600	0.523	5.385
Ileus	0.28	0.40	1.000	0.687	0.214	2.052
Deep wound infection	0.30	1.34	< 0.001	0.226	0.085	0.518
Bleeding	0.66	2.70	< 0.001	0.243	0.129	0.430
Seroma	1.94	5.12	< 0.001	0.379	0.262	0.540
Wound healing disorders	0.30	2.90	< 0.001	0.104	0.041	0.225

*Corrected according to Bonferroni: intraoperative complications (factor 8), postoperative complications (factor 6)

On the contrary, a significant deviation was found to the disadvantage of the laparoscopic IPOM technique concerning the rate of intraoperative complications [lap. IPOM 2.3% vs. sublay 1.3%; OR 1.840, 95% CI (1.290–2.651); *p* ≤ 0.001] (Table 1), mainly bleeding, bowel, and other organ injuries (Table 2).

Main hospital stay showed again advantages for laparoscopic IPOM compared to open sublay with 4.35 ± 3.32 days versus 6.14 ± 5.29 days (*p* < 0.001).

A subgroup analyses of 339 matched pairs with laparoscopic IPOM and open sublay repair of incisional hernias with defect size ≥ 10 cm was also performed. The only significant disadvantage of the open sublay repair was found to be the postoperative complication rate [lap. IPOM 5.0% vs. sublay 18%; OR 0.279, 95% CI (0.153–0.483); *p* < 0.001] and the complication-related reoperation rate [lap IPOM 2.1% vs. sublay 7.7%; OR 0.269, 95% CI (0.099–0.637); *p* = 0.001] (Table 3). No significant deviation in the recurrence rate to the disadvantage for laparoscopic IPOM was identified.

**Table 3:** Results of matched pairs analyses in percentage for the subgroup of patients with incisional hernia with defect size W3 ≥ 10 cm

	Disadvantages		p-Value	OR for matched samples		
	Lap. IPOM	Open sublay		OR		
Intraoperative complication	3.54	2.36	0.503	1.500	0.564	4.230
General complication	5.01	4.13	0.720	1.214	0.563	2.661
Postoperative complication	5.01	17.99	< 0.001	0.279	0.153	0.483
Reoperation	2.06	7.67	0.001	0.269	0.099	0.637
Recurrence on 1-year follow-up	5.60	4.72	0.736	1.187	0.578	2.469
Pain on exertion on 1-year follow-up	15.34	13.57	0.614	1.130	0.746	1.719
Pain in rest on 1-year follow-up	11.21	9.44	0.550	1.187	0.722	1.963
Pain requiring treatment on 1-year follow-up	7.37	5.60	0.451	1.316	0.696	2.527

In comparison to the outcome of the total patient population, this subgroup with larger defect sizes demonstrates higher perioperative complication, chronic pain, and recurrence rates for both surgical techniques.

## Discussion

When comparing laparoscopic IPOM and open sublay approaches in the repair of incisional hernias, the current PS matching analysis of prospective data obtained from the Herniamed Hernia Registry identified no difference in the proportion of patients experiencing chronic pain or recurrence after 1-year follow-up. However, to its disadvantage, the laparoscopic IPOM technique was found to be associated with significantly increased rates of intraoperative complications, particularly bleeding, bowel, and other organ injuries. In discrepancy to the literature, this registry study does not demonstrate a higher rate of bowel, but of total organ injuries in the laparoscopic IPOM group. On the other hand, patients operated on with the open sublay approach experienced significantly higher rates of surgical postoperative complications, predominantly surgical site infection, seroma, and bleeding combined with higher rates of complication-related reoperations. Furthermore, rates of general postoperative complications were also observed to be increased with the open sublay technique. Additionally, the hospital stay is significantly longer for the open sublay technique.

Therefore, in the context of a relatively large population of patients found in everyday clinical routine, the current analysis confirms the findings of the meta-analyses mentioned previously [13–16]. Beyond that, this study establishes a direct comparison between the best open technique, the sublay operation [2], and the laparoscopic IPOM. Furthermore, this analysis again clearly demonstrates that intraoperative complications, namely, bleeding, bowel, and other organ injuries are the Achilles Heel of the minimally invasive approach. Consequently, the expertise of the surgeon and rigorous adherence to guidelines are of paramount importance in the prevention of intraoperative complications [17–21].

The major disadvantage of the open sublay technique is the highly significantly increased rate of surgical site infection, seroma, and bleeding, which is closely associated with the requirement to reoperate.

In accordance with these findings, the laparoscopic IPOM technique should be favored over the open sublay approach in the repair of incisional hernias, given that surgical expertise is evident.

However, the international guidelines state that the recurrence rate after laparoscopic IPOM repair increases when the defect size exceeds 10 cm. Thus it is outlined that laparoscopic IPOM is no longer indicated under those circumstances [17–21]. As of yet, the roles of additional defect suturing and proper mesh overlap in those cases cannot be assessed due to the insufficient quality of existing studies [30, 31] as well as the scarcity of long-term data [17–21]. Our subgroup analysis of 339 matched pairs of laparoscopic IPOM and sublay technique with defect sizes ≥ 10 cm demonstrates no significant difference in the recurrence rate with 5.6% versus 4.7% (*p* = 0.736), but the relatively low number of cases and the short follow-up of only 1 year needs to be considered.

Incorrect or missing data limit a registry [27]. In the Herniamed Hernia Registry, all participating surgeons or responsible chairmen of surgical departments sign a contract for data correctness and completeness. Missing data are indicated by the registry software. Postoperative outcomes are once again reviewed at 1-year follow-up. As part of the certification process of hernia centers, data entry can be controlled by experts. The best safeguard is to match the data against another registry, administrative data, and/or the literature [32]. Voluntary participation in the registry is another limitation of this study due to the possibility of no inclusion of cases with complication.

A further limitation represents the use of different meshes, different mesh fixation, and different techniques.

The findings presented here are mainly in concordance with the existing meta-analyses [13–16] and the statements and recommendations of the international Guidelines [17–21]. Also 5-year follow-up data of the Danish Hernia Database showed no disadvantages in terms of recurrence rate and mesh-related complications for laparoscopic IPOM compared to open incisional hernia repair [33]. Another study reported an incidence of intestinal obstruction secondary to adhesions in 11.5% [34]. The cost of surgery is higher for laparoscopic procedure, but a shorter hospital stay may make laparoscopic surgery more cost effective [18]. More data on the outcome of incisional hernia repair in laparoscopic IPOM technique with defect size W3 ≥ 10 cm according to the EHS classification are urgently needed.

Innovations like the mini-or less-open sublay (MILOS) and EMILOS techniques seem to improve the outcome of the sublay procedure leading to advantages in comparison to laparoscopic IPOM [35, 36].

In summary, the PS matching analysis of data from the Herniamed Hernia Registry presented here for comparison of laparoscopic IPOM vs open sublay in incisional hernia repair demonstrates clear advantages for the minimally invasive technique regarding postoperative surgical and general complications as well as complication-related reoperations, but disadvantages concerning intraoperative complications, mainly bleeding, bowel, and other organ injuries. No significant difference can be found in recurrence and pain rates after 1-year follow-up. The findings of this registry analysis confirm the validity of the literature data and the statements and recommendations of the international guidelines. Laparoscopic IPOM needs to be more evaluated in incisional hernia repair with defects ≥ 10 cm.

## References

[1] Liang MK, Holihan JL, Itani K (2016). Ventral hernia management. Ann Surg.

[2] Holihan JL, Nguyen DH, Nguyen MT, Mo J, Kao LS, Liang MK (2016). Mesh location in open ventral hernia repair: a systematic review and network meta-analysis. World J Surg.

[3] Pham CT, Perera CL, Watkin DS, Maddern GL (2009). Laparoscopic ventral hernia repair: a systematic review. Surg Endosc.

[4] Forbes SS, Eskicioglu C, McLeod RS, Okrainec A (2009). Meta-analysis of randomized controlled trials comparing open and laparoscopic ventral and incisional hernia repair with mesh. Br J Surg.

[5] Sajid MS, Bokhari SA, Mallick AS, Cheek E, Baig MK (2009). Laparoscopic versus open repair of incisional/ventral hernia: a meta-analysis. Am J Surg.

[6] Sauerland S, Walgenbach M, Habermalz B, Seiler CM, Miserez M (2011). Laparoscopic versus open surgical techniques for ventral or incisional hernia repair. Cochrane Database Syst Rev.

[7] Zhang Y, Zhou H, Chai Y, Cao C, Jin K, Hu Z (2014). Laparoscopic versus open incisional and ventral hernia repair: a systematic review and meta-analysis. World J Surg.

[8] Castro PMV, Rabelato JT, Monteiro GGR, del Guerra GC, Mazzurana M, Alvarez GA (2014). Laparoscopy versus laparotomy in the repair of ventral hernia: systematic review and meta-analysis. Arq Gastroenterol.

[9] Köckerling F, Schug-Paß C, Adolf D, Reinpold W, Stechemesser B (2015). Is pooled data analysis of ventral and incisional hernia repair acceptable?. Front Surg.

[10] Kurian A, Gallagher S, Cheeyandira A, Josloff R (2010). Laparoscopic repair of primary versus incisional ventral hernias: time to recognize the differences?. Hernia.

[11] Subramanian A, Clapp ML, Hicks SC, Awad SS, Liang MK (2013). Laparoscopic ventral hernia repair: primary versus secondary hernias. J Surg Res.

[12] Stirler VMA, Schoenmaeckers EJP, de Haas RJ, Raymakers JTFJ, Rakic S (2014). Laparoscopic repair of primary and incisional ventral hernias: the differences must be acknowledged. Surg Endosc.

[13] Al Chalabi H, Larkin J, Mehigan B, McCormick P (2015). A systematic review of laparoscopic versus open abdominal incisional hernia repair, with meta-analysis of randomized controlled trials. Int J Surg.

[14] Awaiz A, Rahman F, Hossain MB, Yunus RM, Khan S, Memon B, Memon MA (2015). Meta-analysis and systematic review of laparoscopic versus open mesh repair for elective incisional hernia. Hernia.

[15] Jensen KK, Jorgensen LN, Awaiz A (2015). Comment to: meta-analysis and systematic review of laparoscopic versus open mesh repair for elective incisional hernia. Hernia 2015 19:449–463. Hernia.

[16] Awaiz A, Rahman F, Hossain MB, Yunus RM, Khan S, Memon B, Memon MA (2005). Reply to comment to Meta-analysis and systematic review of laparoscopic versus open mesh repair for elective incisional hernia. Jensen K, Jorgensen LN. Hernia.

[17] Bittner R, Bingener-Casey J, Dietz U, Fabian M, Ferzli GS, Fortelny RH, Köckerling F, Kukleta J, LeBlanc K, Lomanto D, Misra MC, Bansal VK, Morales-Conde S, Ramshaw B, Reinpold W, Rim S, Rohr M, Schrittwieser R, Simon T, Smietanski M, Stechemesser B, Timoney M, Chowbey P (2014). Guidelines for laparoscopic treatment of ventral and incisional abdominal wall hernias (International Endohernia Society [IEHS])—part 1. Surg Endosc.

[18] Bittner R, Bingener-Casey J, Dietz U, Fabian M, Ferzli GS, Fortelny RH, Köckerling F, Kukleta J, LeBlanc K, Lomanto D, Misra MC, Morales-Conde S, Ramshaw B, Reinpold W, Rim S, Rohr M, Schrittwieser R, Simon T, Smietanski M, Stechemesser B, Timoney M, Chowbey (2014). Guidelines for laparoscopic treatment of ventral and incisional abdominal wall hernias (International Endohernia Society [IEHS])—part 2. Surg Endoc.

[19] Bittner R, Bingener-Casey J, Dietz U, Fabian M, Ferzli G, Fortelny RH, Köckerling F, Kukleta J, LeBlanc K, Lomanto D, Misra M, Morales-Conde S, Ramshaw B, Reinpold W, Rim S, Rohr M, Schrittwieser R, Simon T, Smietanski M, Stechemesser B, Timoney M, Chowbey P (2014). Guidelines for laparoscopic treatment of ventral and incisional abdominal wall hernias (international Endohernia Society [IEHS])—part III. Surg Endosc.

[20] Silecchia G, Campanile FC, Sanchez L, Ceccarelli G, Antinori A, Ansaloni L, Olmi S, Ferrari GC, Cuccurullo D, Baccari P, Agresta F, Vettoretto N, Piccoli M (2015). Laparoscopic ventral/incisional hernia repair: updated guidelines from the EAES and EHS endorsed Consensus Development Conference. Surg Endosc.

[21] Earle D, Roth JS, Saber A, Haggerty S, Bradley JF, Fanelli R, Price R, Richardson WS, Stefanidis D, SAGES Guidelines Committee (2016). SAGES guidelines for laparoscopic ventral hernia repair. Surg Endosc.

[22] Bisgaard T, Kehlet H, Bay-Nielsen MB, Iversen MG, Wara P, Rosenberg J, Friis-Andersen HF, Jorgensen LN (2009). Nationwide study of early outcomes after incisional hernia repair. Br J Surg.

[23] Lonjon G, Porcher R, Ergina P, Fouet M, Boutron (2017). Potential pitfalls of reporting and bias in observational studies with propensity score analysis assessing a surgical procedure. Ann Surg.

[24] Booth CM, Tannock IF (2014). Randomised controlled trials and population-based observational research: partners in the evolution of medical evidence. Br J Canter.

[25] Stechemesser B, Jacob DA, Schug-Paß C, Köckerling F (2012). Herniamed: an internet-based registry for outcome research in hernia surgery. Hernia.

[26] Köckerling F, Simon T, Hukauf M, Hellinger A, Fortelny R, Reinpold W, Bittner R (2017). The importance of registries in the postmarketing surveillance of surgical meshes. Ann Surg.

[27] Köckerling F, Bittner R, Kofler M, Mayer F, Adolf D, Kuthe A, Weyhe D (2017). Lichtenstein versus total extraperitoneal patch plasty versus transabdominal patch plasty technique for primary unilateral inguinal hernia repair. Ann Surg.

[28] Baucom RB, Ousley J, Feurer ID, Beveridge GB, Pierce RA, Holzman MD, Sharp KW, Poulose BK (2016). Patient reported outcomes after incisional hernia repair—establishing the ventral hernia recurrence inventory. Am J Surg.

[29] Muysoms FE, Miserez M, Berrevoet F, Campanelli G, Champault GG, Chelala E, Dietz UA, Eker HH, El Nakadi I, Hauters P, Hidalgo Pascual M, Hoeferlin A, Klinge U, Montgomery A, Simmermacher RKF, Simons MP, Smietanski M, Sommeling C, Tollens T, Vierendeels T, Kingsnorth A (2009). Classification of primary and incisional abdominal wall hernias. Hernia.

[30] Tandon A, Pathak S, Lyons NJR, Nunes QM, Daniels IR, Smart NJ (2016). Meta-analysis of closure of the fascial defect during laparoscopic incisional and ventral hernia repair. Br J Surg.

[31] LeBlanc K (2016). Proper mesh overlap is a key determinant in hernia recurrence following laparoscopic ventral and incisional ventral and incisional hernia repair. Hernia.

[32] Hannan EL, Cozzens K, King SB, Walford G, Shah NR (2012). The New York state cardiac registries. Coll of Cardiol.

[33] Kokotovic D, Bisgaard T, Helgstrand F (2016). Long-term recurrence and complications associated with elective incisional hernia repair. JAMA.

[34] Tandom A, Shahzad K, Pathak S, Oommen CM, Nunes QM, Smart N (2016). Parietex™ composite mesh versus DynaMesh®-IPOM for laparoscopic incisional and ventral hernia repair: a retrospective cohort study. Ann R Coll Surg Engl:.

[35] Reinpold W, Schröder M, Berger C, Nehls J, Schroeder A, Hukauf M, Köckerling F, Bittner R (2018). Mini- or Less-open Sublay Operation (MILOS): a new minimally invasive technique for the extraperitoneal mesh repair of incisional hernias. Ann Surg.

[36] Schwarz J, Reinpold W, Bittner R (2017). Endoscopic mini/less open sublay technique (EMILOS) a new technique for ventral hernia repair. Langenbecks Arch Surg.

